# Magnetic resonance imaging of skull and brain parameters in fetuses with intrauterine growth restriction

**DOI:** 10.1590/0100-3984.2020.0025

**Published:** 2021

**Authors:** Ronaldo Eustáquio de Oliveira Júnior, Sara Reis Teixeira, Eduardo Félix Martins Santana, Jorge Elias Junior, Fabricio da Silva Costa, Edward Araujo Júnior, Alessandra Cristina Marcolin

**Affiliations:** 1 Department of Obstetrics and Gynecology, Faculdade de Medicina de Ribeirão Preto da Universidade de São Paulo (FMRP-USP), Ribeirão Preto, SP, Brazil.; 2 Department of Medical Imaging, Hematology, and Clinical Oncology, Faculdade de Medicina de Ribeirão Preto da Universidade de São Paulo (FMRP-USP), Ribeirão Preto, SP, Brazil.; 3 Department of Obstetrics, Escola Paulista de Medicina da Universidade Federal de São Paulo (EPM-Unifesp), São Paulo, SP, Brazil.; 4 Department of Perinatology, Hospital Israelita Albert Einstein, São Paulo, SP, Brazil.

**Keywords:** Fetus, Ultrasonography, Magnetic resonance imaging, Intrauterine growth restriction, Brain, Cerebrospinal fluid, Feto, Ultrassonografia, Ressonância magnética, Retardo do crescimento fetal, Encéfalo, Líquido cefalorraquidiano

## Abstract

**Objective:**

To compare fetuses with intrauterine growth restriction (IUGR) and those with normal growth, in terms of skull and brain measurements obtained by magnetic resonance imaging (MRI).

**Materials and Methods:**

This was a prospective cohort study including 26 single fetuses (13 with IUGR and 13 with normal growth), evaluated from 26 to 38 weeks of gestation. Using MRI, we measured skull and brain biparietal diameters (BPDs); skull and brain occipitofrontal diameters (OFDs); corpus callosum length and area; transverse cerebellar diameter; extracerebral cerebrospinal fluid (eCSF); and right and left interopercular distances (IODs).

**Results:**

The following were significantly smaller in IUGR fetuses than in control fetuses: skull BPD (76.9 vs. 78.2 mm; *p* = 0.0029); brain BPD (67.8 vs. 71.6 mm; *p* = 0.0064); skull OFD (93.6 vs. 95 mm; *p* = 0.0010); eCSF (5.5 vs. 8.2 mm; *p* = 0.0003); right IOD (9.8 vs. 13.9 mm; *p* = 0.0023); and left IOD (11.8 vs. 16.3 mm; *p* = 0.0183). The skull BPD/eCSF, brain BPD/eCSF, skull OFD/eCSF, and brain OFD/eCSF ratios were also lower in IUGR fetuses.

**Conclusion:**

IUGR fetuses had smaller OFD and BPD, both skull and brain, and less eCSF when compared to normal growth fetuses.

## Abstract (pt)

**Objetivo:**

Comparar medidas do crânio e encéfalo por meio da ressonância magnética (RM) de fetos com restrição do crescimento intrauterino (RCIU) e com crescimento adequado.

**Materiais e Métodos:**

Realizou-se um estudo de coorte prospectivo com 13 fetos com RCIU e 13 controles entre 26 e 38 semanas. Foram realizadas as seguintes medidas por RM: diâmetro biparietal (DBP) e diâmetro occipitofrontal (DOF) cerebral e ósseo, comprimento e área do corpo caloso (CPC), diâmetro transverso do cerebelo, líquido cerebroespinhal (LCE) extracerebral e distância interopercular (DIO) direita e esquerda.

**Resultados:**

Observaram-se diferenças significativas nas medidas do DBP ósseo (76,9 vs. 78,2 mm; *p* = 0,0029), DBP cerebral (67,8 vs. 71,6 mm; *p* = 0,0064) e DOF ósseo (93,6 vs. 95 mm; *p* = 0,0010) em fetos com RCIU em relação aos fetos com crescimento normal. Observaram-se, ainda, diferenças significativas nas médias do LCE extracerebral (5,5 vs. 8,2 mm; *p* = 0,0003) e DIO direita (9,8 vs. 13,9 mm; *p* = 0,0023) e esquerda (11,8 vs. 16,3 mm; *p* = 0,0183) em fetos com RCIU em relação aos controles. Fetos com RCIU e normais tiveram diferenças entre DBP ósseo/LCE, DBP cerebral/LCE, DOF/LEC, e DOF cerebral/LCE.

**Conclusão:**

Fetos com RCIU tiveram menores DBP e DOF, ambos crânio e encéfalo, e menor LCE extracerebral que fetos com crescimento adequado.

## INTRODUCTION

Intrauterine growth restriction (IUGR), which occurs in 5-10% of pregnancies, is a major cause of perinatal mortality and morbidity, resulting in disorders of psychomotor and neuromotor development, as well as cardiovascular diseases and endocrine disorders in adults^(1)^. The variety of etiologies and the lack of prenatal interventions to prevent or correct growth deficit make the management of IUGR a challenge. The leading cause of IUGR (responsible for 80% of cases) is placental insufficiency, which results in progressive and relatively predictable fetal impairment^(2)^.

The neurological deficits associated with IUGR seem to be the result of brain reorganization, as suggested by studies showing differences between infants with and without IUGR in terms of brain metabolism, morphology, and connections, as well as neurological microstructure^(3,4)^. Although ultrasound is the primary modality for evaluating the fetus, ultrasound examinations have limited ability to detect these types of abnormalities. Fetal magnetic resonance imaging (MRI) adds information to ultrasound examinations and is a highly accurate method for the early detection, confirmation, or exclusion of suspected changes. It has been used in order to estimate fetal brain oxygenation or to evaluate brain changes due to IUGR^(5,6)^. Some MRI-based studies have shown that IUGR neonates have reduced gray matter and hippocampal volumes, as well as significant delays in cortical development, with conflicting patterns of gyration and sulcation^(7,8)^. In a recent study, Kyriakopoulou et al.^(9)^ quantified the brain growth of normal fetuses throughout the second half of pregnancy using two-dimensional and three-dimensional biometric parameters on MRI scans. Thus, the cranial MRI scans of IUGR fetuses could be compared with those of normal fetuses, which would improve knowledge of the neurodevelopmental patterns associated with fetal malnutrition. Therefore, MRI may be an auxiliary method for diagnosing neurological lesions associated with chronic fetal hypoxia and for the early identification of fetuses at high risk for future neurological impairment. However, the few MRI studies of IUGR fetuses have limited clinical applicability, especially because of the long acquisition time and complex image processing, which are often affected by fetal movement. Therefore, the objective of this study was to compare the MRI measurements of the skull and brain obtained in IUGR fetuses with those obtained in normal fetuses.

## MATERIALS AND METHODS

### Study sample

This was a prospective cohort study including singleton IUGR fetuses (IUGR group) and normal fetuses (control group), all evaluated from week 26 to week 38 of gestation. The control group comprised one healthy fetus for each week of gestation (i.e., weeks 26, 27, 28, 29, 30, 31, 32, 33, 34, 35, 36, 37, and 38), the pregnant women having been randomly selected from the database of our institution, and the IUGR group comprised one IUGR fetus for each respective week of gestation, the diagnosis of IUGR having been established during ultrasound examinations of the pregnant women. Therefore, the fetus pairs were matched for gestational age (GA), as calculated from the date of the last menstrual period and confirmed by ultrasound in the first trimester. In the control and IUGR groups, the MRI examination was performed within three days after the ultrasound. All of the pregnant women were recruited from among those screened in 2016 at the Hospital das Clínicas of the University of São Paulo at Ribeirão Preto Medical School. This study was approved by the Research Ethics Committee of the Hospital das Clínicas (Reference no. 13840/2015), and all participants gave written informed consent.

The control group included pregnant women referred for MRI after ultrasound, according to defined clinical criteria such as maternal diseases, suspected fetal congenital anomalies, obesity preventing adequate fetal ultrasound evaluation, and suspected placental diseases^(10)^. In the control group, birth weights were appropriate for GA, defined as being between the 10th and 90th percentiles^(11)^, and the umbilical artery (UA) Doppler ultrasound findings were normal^(12)^.

The IUGR group included pregnant women in whom no fetal structural abnormalities were identified and a diagnosis of IUGR was made on the basis of the consensus criteria established by Gordijn et al.^(13)^: an estimated fetal weight (EFW) below the 3rd percentile or between the 3rd and 10th percentiles^(11)^; and a UA resistance index (RI) above the 95th percentile^(12)^, with or without a cerebroplacental ratio (CPR) < 1^(14)^. Pregnant women in whom there were major structural or chromosomal fetal abnormalities diagnosed in the neonatal period were excluded, as were those who were lost to follow-up and those who had claustrophobia severe enough to preclude MRI. All of the women were followed to delivery. Apgar scores, hospitalizations in neonatal intensive care units, and adverse perinatal outcomes were recorded.

### Ultrasound

The same ultrasound system (Voluson E8 Expert; General Electric Medical Systems, Milwaukee, WI, USA) was used in all ultrasound evaluations. The following biometric parameters were measured: biparietal diameter (BPD), occipitofrontal diameter (OFD), head circumference (HC), femur length, abdominal circumference (AC), cephalic index^(15)^, and EFW^(16)^. The single deepest pocket of amniotic fluid was measured and considered abnormal if below the 5th percentile for the GA^(17)^. Doppler ultrasound of the UA was obtained at the level of umbilical cord insertion into the placenta and was considered abnormal if the RI was above the 95th percentile^(12)^. Doppler ultrasound of the middle cerebral artery was obtained at the circle of Willis after its origin from the internal carotid artery, and an RI below the 5th percentile was considered abnormal^(12)^. The CPR was calculated as the ratio between the RI of the middle cerebral artery and that of the UA, the ratio being considered abnormal if < 1. Doppler ultrasound of the ductus venosus was obtained in the longitudinal plane of the fetal upper abdomen, beginning at the umbilical portion of the portal vein, a pulsatility index above the 90th percentile being considered abnormal^(18)^.

### MRI

The MRI scans were acquired in a 3.0-T scanner (Achieva; Koninklijke Philips Electronics N.V., Eindhoven, The Netherlands). A 16-channel body coil was positioned anteriorly over the maternal abdomen and centered on the fetal brain, which was evaluated in the coronal, sagittal, and axial planes with single-shot turbo spin-echo T2-weighted sequences, with additional axial T1- and T2-weighted fat-saturated gradient-echo and diffusion-weighted sequences. All MRI scans were saved as Digital Imaging and Communications in Medicine format files in the Picture Archiving and Communication System and subsequently transferred to a workstation (OsiriX MD; https://www.osirix-viewer.com/osirix/overview/). Linear dimensions were calculated from images reconstructed from sequential MRI slices, including brain BPD and OFD; skull BPD and OFD; HC; corpus callosum length and area; pons width and height; transverse cerebellar diameter; cerebellar vermis height, width, and area; left and right atrial diameters; extracerebral cerebrospinal fluid (eCSF); and left and right axial and coronal interopercular distances (IODs).

**Figure 1:**
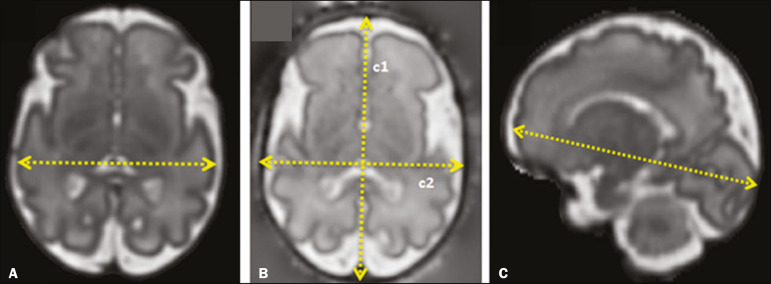
MRI measurements of brain BPD (**A**), skull BPD (**B**) and brain OFD (**C**).

**Figure 2:**
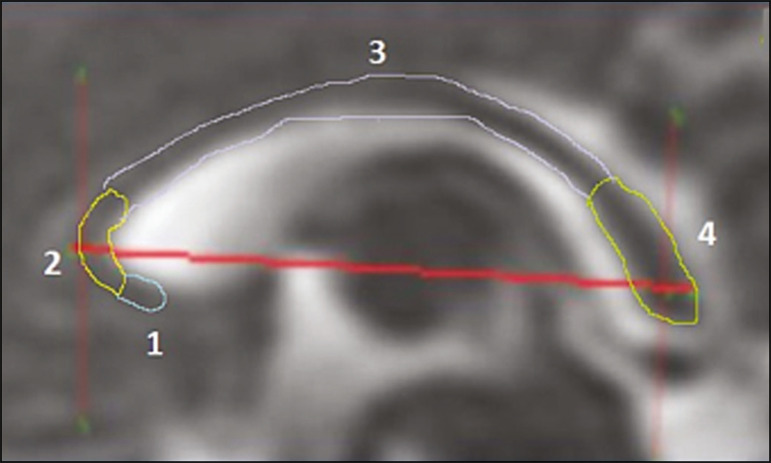
MRI of the corpus callosum. The red line represents the measurement of its length. 1, rostrum; 2, knee; 3, body; 4, splenium.

**Figure 3:**
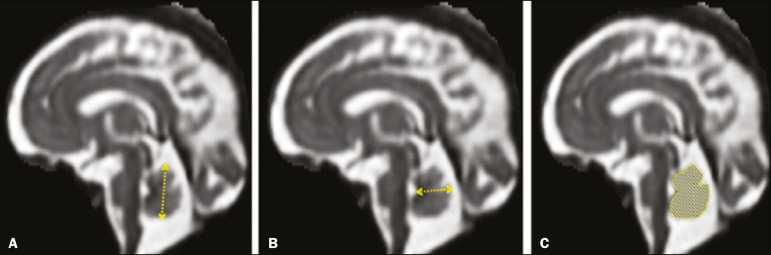
MRI measurements of cerebellar vermis height (**A**), width (**B**), and area (**C**).

Skull and brain BPDs were measured in the transverse plane^(9)^, the former being measured at the maximum width of the brain and the latter corresponding to the largest transverse diameter of the fetal skull. Skull and brain OFDs were measured in the sagittal plane, the former corresponding to the distance between the extremities of the frontal and occipital lobes, whereas the latter corresponded to the maximum distance between the frontal and occipital cranial bones. The HC was calculated by using the equation $1.62\times \left(\mathrm{skull}\;\mathrm{BPD}+\mathrm{skull}\;\mathrm{OFD}\right)$, as illustrated in Figure 1. The corpus callosum was identified in the mid-sagittal plane as a hypointense curved structure. The evaluation plan included identification of the cavum septum pellucidum, thalamus, mesencephalon, cerebellar vermis, and cisterna magna^(19)^, as depicted in Figure 2. The pons was evaluated in the mid-sagittal section of the fetal skull, the height and width of the pons corresponding to the largest longitudinal and anteroposterior diameters, respectively. Cerebellar diameter was defined as the largest lateral diameter in the transverse plane. The height, width, and area of the cerebellar vermis were measured in the mid-sagittal plane, the height and width corresponding to the largest longitudinal and anteroposterior diameters, respectively. The area of the cerebellar vermis was outlined and calculated using the OsiriX MD software design tool^(9)^, as shown in Figure 3. The atrial diameter was measured bilaterally. The eCSF was measured by using the following formula^(9)^:

eCSF=skullBPD−brainBPD

**Figure 4:**
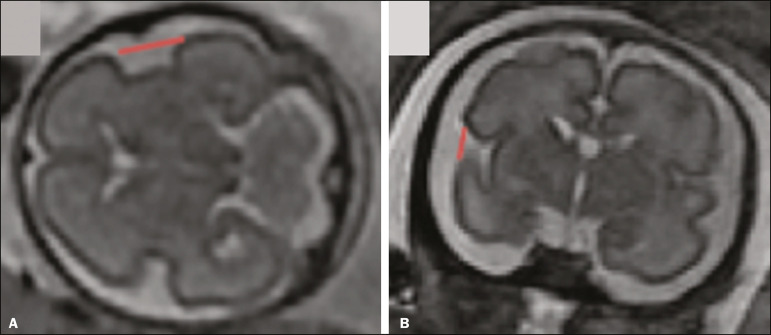
MRI measurements of anteroposterior IOD (**A**) and craniocaudal IOD (**B**).

The left and right anteroposterior (axial) IODs were measured in the transverse plane of the fetal skull and corresponded to the distances between the anterior and posterior edges, respectively, of the Sylvian fissure in its outer brain portion, at the level of the 3rd ventricle. The left and right craniocaudal (coronal) IODs were measured in the coronal plane of the fetal skull and corresponded to the distances between the upper and lower edges, respectively, of the Sylvian fissure in its outer brain portion, also at the level of the 3rd ventricle (Figure 4).

### Statistical analysis

The data were analyzed with the Statistical Package for the Social Sciences, version 13.0 (SPSS Inc., Chicago, IL, USA). The primary outcome measures were the dimensions of the brain structures. Secondary outcome measures were the Apgar scores, hospitalization in a neonatal intensive care unit, adverse perinatal outcomes (intraventricular hemorrhage, periventricular leukomalacia, hypoxic-ischemic encephalopathy, necrotizing enterocolitis, bronchopulmonary dysplasia, sepsis, and neonatal death). In both groups, differences between variables were analyzed with the Wilcoxon test (for continuous variables) or the chi-square test (for categorical variables). Values of *p* < 0.05 were considered statistically significant.

## RESULTS

A total of 78 pregnant women were enrolled. Of those 78 women, 52 were excluded from the analysis, for the following reasons: failure to appear for the MRI (n = 6); claustrophobia (n = 16); missing data in medical records (n = 9); having delivered before the MRI (n = 18); and major fetal structural anomalies having been detected on MRI (n = 3). Therefore, the final sample comprised 26 pregnant women (13 in each group). Demographic variables of the women are shown in Table 1. With the exception of maternal age, no significant differences were observed between the IUGR and control groups.

**Table 1:** Baseline characteristics of pregnant women with IUGR fetuses and normal (control) fetuses.

Maternal variable	Group		*P*
	IUGR	Control	
Age (years), median (range)	27.0 (21.0-34.0)	30.0 (25.0-39.0)	0.0423
Ethnicity, n (%)			
White	10 (76.92)	10 (76.92)	
Other	3 (23.08)	3 (23.08)	1.000
Marital status, n (%)			
Steady partner	9 (69.23)	10 (76.92)	
No partner	4 (30.77)	3 (23.08)	0.6584
Employed, n (%)			
Yes	8 (61.54)	9 (69.23)	
No	5 (38.46)	4 (30.77)	0.6802
Weight (kg), median (range)	67.5 (52.4-90.0)	73.5 (57.4-92.1)	0.1998
Height (m), median (range)	1.57 (1.48-1.70)	1.65 (1.52-1.69)	0.0219
BMI (kg/m^2^), median (range)	25.4 (22.2-33.6)	27.0 (22.4-34.8)	0.5726
Smoking, n (%)	2 (15.38)	0 (0)	0.48
Diseases, n (%)	7 (53.85)	3 (23.08)	0.2262
Parity, n (%)			
Primigravida	7 (53.85)	4 (30.77)	
Secundigravida	2 (15.38)	6 (46.15)	0.2275
Multigravida	4 (30.77)	3 (23.08)	

BMI, body mass index.

Table 2 shows the fetal ultrasound variables in the IUGR and control groups. The EFW, BPD, and AC dimensions were significantly smaller in the IUGR group than in the control group. In addition, among the fetuses in the IUGR group, a higher-than-normal UA RI was observed in 61.5% and a lower-than-normal CPR was observed in 53.8%, whereas neither was observed in any of the fetuses in the control group. No difference was observed between the two groups in terms of the ductus venosus pulsatility index. An abnormal single deepest pocket of amniotic fluid, was seen in 38.5% of the IUGR fetuses.

**Table 2:** Ultrasound parameters in IUGR fetuses and normal (control) fetuses.

Fetal ultrasound parameter	Group		*P*
	IUGR	Control	
EFW (g), median (range)	1122 (506-2210)	1630 (1100-3027)	0.0402
BPD (cm), median (range)	7.5 (5.8-8.3)	7.8 (6.3-8.8)	0.1729
OFD (cm), median (range)	9.5 (7.7-11.1)	9.7 (7.9-11.5)	0.1169
CI, median (range)	78.1 (72.8-87.0)	77.1 (70.9-84.4)	0.7194
AC (cm), median (range)	22.5 (17.7-28.9)	27.1 (21.7-33.3)	0.0183
UA RI, median (range)	0.77 (0.48-1.0)	0.57 (0.45-0.70)	0.0292
Abnormal UA RI, n (%)	8 (61.5)	0 (0.0)	0.0007
MCA RI, median (range)	0.75 (0.60-0.85)	0.80 (0.70-0.86)	0.0506
Abnormal MCA RI, n (%)	1 (7.7)	0 (0.0)	0.3078
CPR, median (range)	0.97 (0.74-1.70)	1.37 (1.11-1.82)	0.0071
Abnormal CPR, n (%)	7 (53.8)	0 (0.0)	0.002
DV pulsatility index, n (%)	0.40 (0.33-0.93)	0.58 (0.50-0.72)	0.3823
SDP AF (cm), median (range)	3.0 (1.3-7.9)	4.5 (3.4-6.2)	0.0766
Abnormal SDP AF, n (%)	5 (38.5)	0 (0.0)	0.0128

CI, cephalic index; MCA, middle cerebral artery; DV, ductus venosus; SDP AF, single deepest pocket of amniotic fluid.

Table 3 shows the variables obtained through the fetal MRIs. There were significant differences between the IUGR and control fetuses in terms of the skull and brain BPDs, as well as the skull OFD. The brain BPD/cerebellum ratio tended to be lower in IUGR fetuses than in control fetuses (*p* = 0.054). There were also significant differences in terms of the eCSF measurements, the eCSF being below the 10th percentile present in nine (69.2%) of the IUGR fetuses compared with only two (15.4%) of the control fetuses, as well as in terms of the skull BPD/eCSF, brain BPD/eCSF, skull OFD/eCSF, and brain OFD/eCSF ratios, all of which were lower in the IUGR fetuses. Left and right axial IODs were smaller in the IUGR fetuses.

**Table 3:** MRI variables of IUGR fetuses and normal (control) fetuses.

Fetal MRI variable	Group		*P*
	IUGR Median (range)	Control Median (range)	
HC (cm)	27.6 (22.0-31.6)	28.0 (23.4-32.4)	0.2814
HC (%)	1.0 (1.0-26.6)	30.6 (5.4-99.0)	0.0001
Skull BPD (mm)	76.9 (59.2-88.6)	78.2 (63.6-94.0)	0.0029
Skull BPD (%)	1.0 (1.0-22.8)	40.9 (11.2-99.0)	0.0001
Brain BPD (mm)	67.8 (53.5-82.5)	71.6 (56.4-86.7)	0.0064
Brain BPD (%)	6.0 (1.0-85.3)	40.4 (24.9-99.0)	0.0006
Skull BPD/cerebellum	76.9 (59.2-88.6)	78.2 (66.3-94.0)	0.2701
Skull BPD/eCSF	14.3 (8.1-23.3)	9.8 (6.7-13.4)	0.0029
Brain BPD/cerebellum	1.8 (1.6-2.3)	2.0 (1.7-2.0)	0.0540
Brain BPD/eCSF	13.3 (7.1-22.3)	8.7 (5.7-12.4)	0.0029
Skull OFD (mm)	93.6 (75.7-108.0)	95.0 (78.4-106.3)	0.0010
Skull OFD (%)	1.8 (1.0-38.0)	34.3 (8.9-99.0)	0.0037
Brain OFD (mm)	86.1 (68.0-104.0)	87.5 (71.2-100.0)	0.5902
Brain OFD (%)	1.4 (1.0-42.8)	22.1 (2.1-99.0)	0.0045
Skull OFD/cerebellum	2.5 (2.1-3.0)	2.7 (2.1-2.8)	0.1112
Skull OFD/eCSF	16.8 (9.4-27.9)	11.9 (7.9-15.8)	0.0010
Brain OFD/cerebellum	2.3 (2.0-2.7)	2.5 (2.0-2.6)	0.1583
Brain OFD/eCSF	15.5 (8.9-26.5)	11.1 (7.2-14.5)	0.0015
eCSF (mm)	5.3 (3.7-9.2)	8.2 (6.2-12.1)	0.0003
eCSF(%)	1.0 (1.0-36.0)	35.8 (1.0-81.1)	0.0044
Corpus callosum length (mm)	36.0 (29.0-42.5)	40.0 (32.6-47.0)	0.2087
Corpus callosum area (mm)	85.1 (64.8-155.0)	94.0 (56.7-154.0)	0.4570
Pons width (mm)	11.6 (8.2-14.5)	10.6 (8.4-13.3)	0.4265
Pons height (mm)	11.4 (8.6-13.6)	10.6 (9.7-14.7)	0.7189
Cerebellar diameter (mm)	34.8 (29.6-50.5)	34.1 (28.0-51.2)	0.8175
Cerebellar diameter (%)	14.9 (1.0-92.6)	38.6 (4.0-98.2)	0.2592
Vermis width (mm)	11.6 (7.2-15.4)	11.0 (7.7-15.0)	0.7003
Vermis width (%)	5.1 (1.0-76.8)	20.4 (1.3-90.6)	0.0647
Vermis height (mm)	16.7 (9.5-23.4)	17.6 (13.2-22.0)	0.9182
Vermis height (%)	29.6 (1.0-82.0)	31.1 (5.0-69.8)	0.2584
Vermis area (mm)	183.0 (108.0-293.0)	211.0 (89.0-273.0)	0.6080
Vermis area (%)	15.1 (1.0-76.1)	34.5 (1.4-95.5)	0.0577
Right atrial diameter (mm)	5.4 (3.4-6.6)	5.5 (2.5-7.8)	0.4257
Left atrial diameter (mm)	5.3 (3.0-7.3)	5.5 (2.8-9.1)	0.7778
Right axial IOD (mm)	9.8 (2.6-14.6)	13.9 (4.3-18.3)	0.0023
Left axial IOD (mm)	11.8 (2.6-14.3)	16.3 (5.9-20.2)	0.0183
Right coronal IOD (mm)	2.4 (1.0-6.1)	2.6 (1.4-10.3)	0.5044
Left coronal IOD (mm)	2.2 (1.0-5.0)	2.9 (1.5-9.1)	0.1366

Table 4 summarizes the perinatal outcomes. In the IUGR group, deliveries occurred at a lower GA, in most cases (80%) due to fetal distress. However, the rates of cesarean section were high in the control group because of the indications for MRI and maternal contraindications. In addition, the proportion of newborns admitted to the neonatal intensive care unit was higher in the IUGR group, as was the rate of adverse outcomes (*p* = 0.002). No significant differences were observed between the IUGR and control groups considering Apgar scores.

**Table 4:** Delivery and perinatal outcomes among pregnant women with IUGR fetuses and normal (control) fetuses.

Variable	Group		*P*
	IUGR	Control	
Days from diagnosis to delivery, median	8.3	43.3	0.01
GA at delivery (weeks), median (range)	34 (27-38)	39 (35-40)	0.0005
Mode of delivery, n (%)			
Cesarean section	10 (76.9)	9 (69.2)	0.001
Vaginal	3 (23.1)	4 (30.8)	
Reason for cesarean section, n (%)			
Fetal distress	3 (30.0)	0	
Fetal hemodynamic centralization	5 (50.0)	0	
Hypertension	2 (20.0)	0	*
Placenta accreta	0	4 (44.5)	
Labor dystocia	0	3 (33.3)	
Previous cesarean section	0	2 (22.2)	
Birth weight (kg), median (range)	1.34 (0.54-2.53)	3.10 (2.53-4.19)	< 0.0001
Small for gestational age newborn, n (%)	12 (92.3)	1 (7.7)	0.0002
Fetal gender, n (%)			
Male	5 (38.5)	7 (53.9)	0.4314
Female	8 (61.5)	6 (46.1)	
1-min Apgar score < 7, n (%)	5 (38.5)	2 (15.4)	0.2054
5-min Apgar score < 7, n (%)	1 (7.7)	1 (7.7)	0.9999
Admission to the neonatal intensive care unit, n (%)	7 (53.9)	2 (15.4)	0.0108
Adverse perinatal outcome, n (%)	8 (61.5)	0	0.002

* Insufficient number of cases for the statistical analysis.

## DISCUSSION

The findings of the present study provide a better understanding of possible cranial changes in IUGR fetuses. Maternal age was the only demographic variable that differed between the IUGR and control groups. Odibo et al.^(20)^ found that maternal age > 35 years is an independent risk factor for IUGR. In the present study, we found no significant association between maternal smoking and IUGR, probably because our sample size was relatively small. Hammoud et al.^(21)^ reported that, although maternal smoking had no effect on fetal HC or femur length growth rates, fetal AC growth rates were lower among women who smoked during pregnancy than among those who did not.

We found no significant differences in BPD, as measured with ultrasound, between the IUGR and control groups. However, the BPD percentile was significantly lower for the IUGR fetuses than for the control fetuses. Hasegawa et al.^(22)^ analyzed pregnant women with IUGR fetuses and reported that neurological outcomes were worse when the BPD growth rate was < 40% and the birth weight was < 700 g.

In the present study, fetal MRI showed that skull and brain BPDs were significantly lower in the IUGR group, as were skull OFDs. Batalle et al.^(3)^ used diffusion-weighted MRI scans to evaluate the reorganization of white matter brain connections in one-year-old IUGR infants, trying to correlate their findings with neurodevelopmental outcomes evaluated with the Bayley Scales of Infant and Toddler Development. The authors reported significant differences between the IUGR and control infants, showing a correlation between abnormalities of connectivity and poorer performance in the IUGR infants. Leppänen et al.^(23)^ followed extremely low birth weight infants until two years of age and concluded that intracranial Doppler ultrasound parameters were related to intracranial volume, and that reduced brain volume was associated with abnormal neurological outcomes. Those data are relevant, given that more than half (53.8%) of the IUGR fetuses in our study presented an abnormal CPR. However, the probability of IUGR insults leading to abnormal development of brain structures varies by structure, because no significant differences were seen between the IUGR and control fetuses in terms of the dimensions of the fetal HC, pons, and cerebellum.

This study presented some interesting additional findings related to the IOD and eCSF. Right and left axial IOD measurements were smaller in IUGR fetuses than in the control fetuses. The cerebral operculum contains parts of the frontal, temporal, and parietal lobes that cover the insula and unite to form the sylvian fissure. Egaña-Ugrinovic et al.^(24)^ found that IUGR fetuses showed reduced gray and white matter, in a distribution pattern different than that seen in normal fetuses, with significant reductions in the size of the temporal and insular lobes. Therefore, a reduced axial IOD may be directly related to a reduction in the size of the temporal lobe. CSF is produced by the choroid plexus in the lateral, third, and fourth cerebral ventricles, circulating through the subarachnoid space between the arachnoid mater and the pia mater, and its measurement is predominantly affected by the development of the temporal lobes^(9)^. We identified a significant difference between IUGR and control fetuses in terms of the size of the eCSF. The differences between the two groups in terms of the skull BPD/eCSF, brain BPD/eCSF, skull OFD/eCSF, and brain OFD/eCSF ratios, all of which were lower in the IUGR fetuses, suggesting that IUGR results in a relevant reduction in the CSF. Another hypothesis to explain CSF reduction is the destruction of the blood-brain barrier caused by a hypoxic process, which would allow the passage of a greater quantity of CSF than could be reabsorbed^(25)^.

The strengths of the present study include the facts that we evaluated the fetuses in accordance with strict growth restriction criteria and that we followed the subjects prospectively, as well as that the fetal ultrasound and MRI images were analyzed by specialists in the respective fields. In addition, the technique described here has the potential to be a simpler method to evaluate growth-restricted fetuses. The main limitation of our study was the relatively small sample size. Consequently, studies involving larger samples should be conducted before this technique is incorporated into clinical practice.

In summary, the skull BPD, brain BPD, skull OFD, brain OFD, HC, eCSF, and axial IOD are all smaller in IUGR fetuses. There is a need for further studies evaluating the impact that those aspects have on the psychomotor and neuromotor development of IUGR children.
